# Impact of Drought, Salinity, and Their Combination on Growth, Mineral Content, and Plant Secondary Metabolites of Tomatoes ( *Solanum lycopersicum* L.)

**DOI:** 10.1111/ppl.70725

**Published:** 2025-12-30

**Authors:** Niken Ayu Permatasari, Tobias Pöhnl, Susanne Neugart

**Affiliations:** ^1^ Division of Quality and Sensory of Plant Products, Department of Crop Sciences Georg‐August‐Universität Göttingen Göttingen Germany; ^2^ Department of Agroindustrial Technology, Faculty of Agricultural Technology IPB University Bogor Indonesia; ^3^ Department of Food Technology & Nutrition MCI | the Entrepreneurial School Innsbruck Austria

**Keywords:** antioxidant activity, lycopene, minerals, phenolic compounds, yield

## Abstract

Drought and salinity are significant challenges to tomato production under climate change. A 2‐year experiment (2023–2024) with *Solanum lycopersicum* cv. Resi evaluated the effects of drought (25%, 12.5%, and 6.25% of soil weight) and salinity (0.5% and 1.0% NaCl), applied individually and in combination, on yield, mineral uptake, and secondary metabolism. Drought reduced yield by 28%, salinity by 17%, and their combination by 27%. Moderate drought and salinity increased potassium (K^+^) uptake, whereas severe stress reduced calcium (Ca^2+^) concentration and disrupted overall ionic homeostasis. Lycopene and *β*‐carotene decreased under combined stress, whereas chlorogenic acid and naringenin chalcone increased, indicating enhanced antioxidant metabolism. Antioxidant activities (TEAC, DPPH, and TPC) rose under moderate stress, particularly in the warmer 2024 season. Correlation analysis showed that magnesium (Mg^2+^) accumulation was positively associated with antioxidants and carotenoids, supporting redox balance under stress conditions. Overall, these findings indicate that tomato adaptation to drought and salinity relies on coordinated ionic regulation and antioxidant adjustments, both influenced by environmental conditions.

## 1 Introduction

Climate change and global warming intensify drought and salinity stress, which are major constraints on crop productivity worldwide (Moore et al. 2017; Wang et al. 2003; Yang et al. 2021). These abiotic stresses frequently co‐occur, particularly in semi‐arid regions where limited freshwater resources force growers to rely on brackish water for irrigation. These conditions not only reduce yield but also affect the nutritional and functional quality of crops, such as tomatoes, which are widely consumed for their health‐promoting properties (Biondi et al. 2025; Massaretto et al. 2018). Tomatoes (*Solanum lycopersicum L*.) are one of the world's most important horticultural crops, with global production reaching 262 million tons in 2023 (FAOSTAT 2025). Tomato fruits are rich in minerals, carotenoids such as lycopene and *β*‐carotene, and phenolic compounds that contribute to antioxidant capacity and consumer health benefits (George et al. 2004; Martí et al. 2018). However, tomato productivity and quality are highly sensitive to water deficit and salt accumulation in soils (Munns and Tester 2008; Van Zelm et al. 2025). While previous studies (e.g., Botella et al. 2021; Ruggiero et al. 2022) have examined the individual and combined effects of drought and salinity on tomato growth, yield, and certain biochemical traits, a critical gap remains: few have provided an integrated, detailed biochemical profiling of both mineral and secondary metabolite composition, especially across multiple growing seasons. Previous studies have started to investigate the effects of drought and salinity on tomato yield and various metabolites. However, they do not provide a detailed multi‐seasonal analysis of mineral and antioxidative responses alongside detailed morphological traits, which limits our overall understanding of how crops adapt to stress. This integration is essential, as secondary metabolites like carotenoids and phenolics are not only quality determinants but also serve crucial protective roles in stress adaptation (Nisar et al. 2015; Sánchez‐Rodríguez et al. 2010). To address this gap and advance our understanding, we examined the effects of drought, salinity, and their combination on growth, mineral accumulation, and key secondary metabolites in the cocktail tomato cultivar ‘Resi’, a genotype valued for its aromatic flavor and extended harvest period across two growing seasons. We hypothesized that drought would have a stronger impact than salinity. However, moderate stress combinations might induce specific adaptive responses in mineral uptake and secondary metabolism. By linking morphological traits, mineral dynamics, and antioxidant activity, this study provides new insights into stress adaptation and offers implications for maintaining crop quality in the face of climate‐related challenges.

## 2 Materials and Methods

### 2.1 Plant Material and Growth Conditions

This experiment was conducted over two consecutive seasons (2023–2024) at the University of Göttingen (51.54° N, 9.94° E) using the cocktail tomato cv. Resi (Samen Maier, Austria). Seeds were sown in mid‐April in peat‐based trays (Hawita Sowing Soils) and transplanted after 2 weeks into 1 L pots containing organic tomato soil (Tomato and Vegetable Soil CompoSana). Seedlings were grown in a climate chamber (22/18°C day/night; 16‐h photoperiod; 155.19 μmol m^−2^ s^−1^ light; 49%–62% RH). From late May, plants were transferred outdoors into 6 L Mitscherlich pots filled with the same soil supplemented with 100 g long‐term tomato fertilizer per 20 L of soil (Compo). Plants were supported on strings and maintained under natural light and temperature. Environmental conditions (temperature, precipitation) were recorded by a local weather station (see Figures S1 and S2). Pots were covered to minimize evaporation, and control plants were irrigated with fresh water (2.0 μS cm^−1^ at 25°C) as needed to maintain soil moisture close to field capacity (25% of soil weight).

### 2.2 Experimental Treatments, Sample Harvesting, and Processing

**FIGURE 1:**
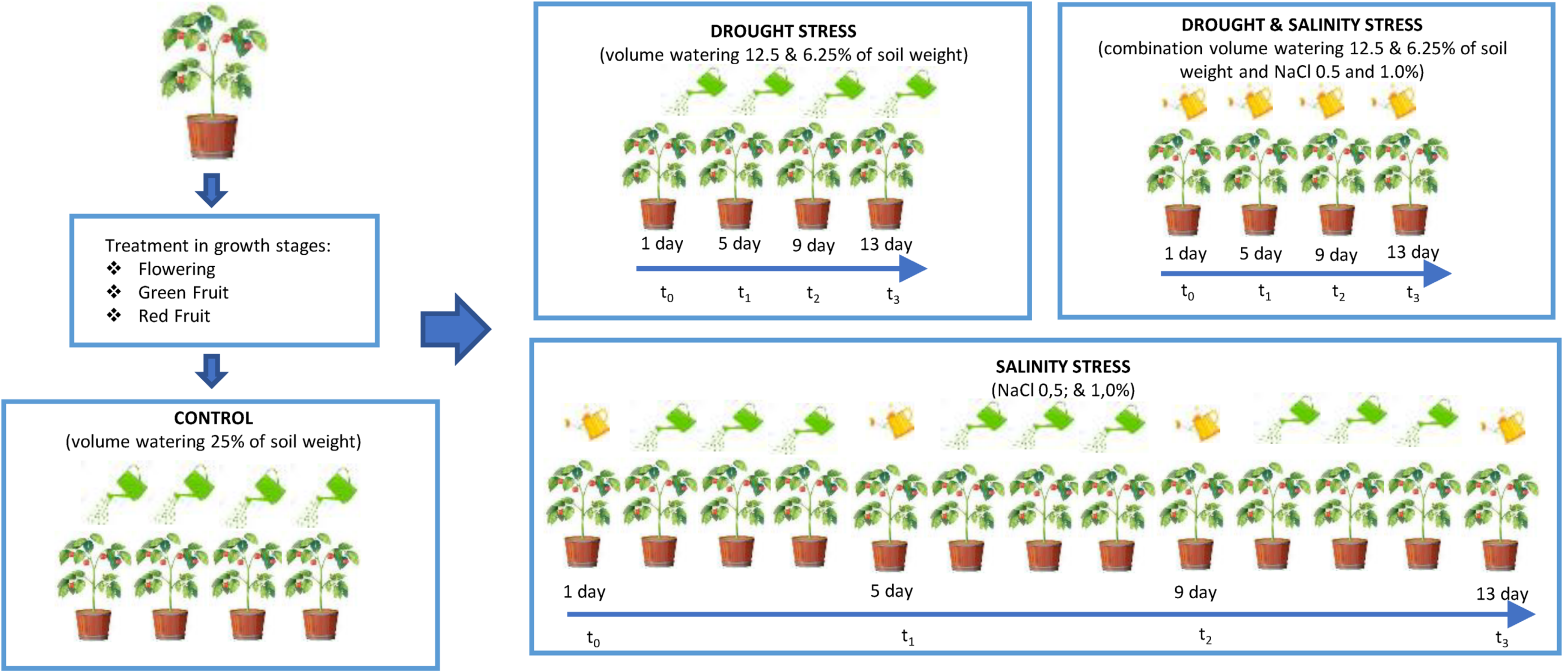
The application of drought, salt, and their combined treatments in tomatoes. Schematic overview of the application of drought (25%, 12.5%, and 6.25% of soil weight), salinity (0.5% and 1.0% NaCl), and their combinations across the main fruit developmental stages during the two growing seasons (2023–2024).

The experiment followed a factorial randomized complete block design with two factors, drought intensity and salinity level, each with four replications. Each replication consisted of three plants in 2023 and five plants in 2024. The increased number of plants in 2024 was to ensure sufficient fruit yield for complementary sensory analyses, which required a larger sample size. Drought stress was applied as three irrigation levels representing soil water content percentages relative to pot weight: well‐watered or control (25% of soil weight), moderate (12.5% of soil weight), and severe drought stress (6.25% of soil weight). Salinity stress was induced by irrigation with NaCl solutions of 0.5% (~86 mM) and 1.0% (~172 mM; Carl Roth), applied either individually or in combination with drought treatments. Stress was applied throughout three fruit developmental stages (flowering, green fruit, and red fruit) over 13 days each (Figure 1). Morphological parameters collected included plant height, stem diameter, number of leaves and fruits, yield per plant, and average fruit weight. Fruits were harvested at the red ripe stage (18–21 weeks after sowing), with six to eight fruits per plant freeze‐dried (EPSILON 2–40, Martin Christ), ground to a fine powder, and stored for analysis.

### 2.3 Determination of Mineral Content

Mineral content analysis was performed following Koch et al. (2019). Briefly, 100 mg of dried sample was digested with 4 mL of HNO_3_ (≥ 65% p.a. ISO) and 2 mL of H_2_O_2_ (30% p.a. IOS stabilized; Carl Roth) using microwave digestion (200°C, 15 bar, 80 min). The digests were diluted to a final volume of 25 mL and analyzed by inductively coupled plasma‐optical emission spectrometry (ICP‐OES; ICAP 7000 Series, Thermo Scientific) at the Albrecht‐Haller Institute of the Georg‐August University Göttingen, Germany. Measurements were done in duplicates.

### 2.4 Determination of Carotenoids

Carotenoid analysis was followed by established protocols (Bayer et al. 2022; Laurenčíková et al. 2023; Neugart et al. 2017). Freeze‐dried samples (5 mg) were extracted with a 1:1 (v/v) mixture of methanol and tetrahydrofuran (MeOH: THF; Carl Roth) and shaken for 10 min in a thermomixer (Eppendorf) at 1400 rpm and 20°C. After centrifugation (9000 g, 5 min, 20°C), the pellets were re‐extracted twice, and the combined supernatants (~1500 μL) were evaporated to dryness using an RVC 2‐25 CD plus rotary evaporator (Martin Christ) with a PC 3001 Vario select pump (Vacuubrand). The residue was re‐dissolved in 100 μL MTBE and 150 μL MeOH (Carl Roth), shaken (800 rpm, 5 min, 20°C), filtered through a 0.2 μm PTFE syringe filter (Chromafil; Macherey‐Nagel), and stored at −80°C until analysis. Carotenoids were quantified on a Shimadzu Prominence HPLC system (Shimadzu), which included an LC‐20AT pump, a CTO‐10AS column oven, a DGU‐20AS degasser, and an SPD‐M20A UV/visible diode array detector. Quantification was based on external calibration curves (*R* ^2^ > 0.99) prepared from authentic standards (Carl Roth) at 474 nm (lycopene) and 450 nm (*β*‐carotene, lutein). All extraction protocols were performed in duplicates for reproducibility. Chromatographic purity was regularly monitored to minimize interference, and the quantification results included standard deviations to reflect data variability and method reliability.

### 2.5 Determination of Phenolic Compounds

Phenolic compounds were analyzed following established protocols (Bayer et al. 2022; Laurenčíková et al. 2023; Neugart et al. 2017). Freeze‐dried sample (50 mg) was extracted with 1.5 mL MeOH:H_2_O: FA (60:39.5:0.5 v/v/v; MeOH HPLC‐grade and FA from Carl Roth) using a ThermoMixer (Eppendorf) at 2000 rpm and 20°C for 40 min. The extracts were centrifuged at 9000 *g* for 5 min at 20°C, and the pellets were re‐extracted twice. The combined supernatants were adjusted to 5 mL, centrifuged again (2250 g, 5 min, 20°C), and then filtered through a 0.2 μm RC syringe filter (RC‐20/13 MS, Macherey–Nagel). Extracts were stored at −20°C until analysis. Phenolics were quantified using the Shimadzu Prominence HPLC system described above. Detection wavelengths were 330 nm (chlorogenic acid) and 370 nm (quercetin‐3‐rutinoside, naringenin chalcone). Quantification was performed against external calibration curves (*R* ^2^ > 0.99) prepared from authentic standards (Carl Roth). All extractions and analyses were performed in duplicates to ensure reproducibility, and chromatographic purity was routinely checked to avoid overlapping peaks.

### 2.6 Determination of Antioxidant TEAC, DPPH, and TPC Assays

Antioxidant activity was assessed photometrically by the Total Phenolic Content (TPC) assay using Folin–Ciocalteu phenol reagent (Merck), the Trolox Equivalent Antioxidant Capacity (TEAC) assay using ABTS (2,2́–azino‐bis (3‐ethylbenzothiazoline‐6‐sulfonic acid) diammonium salt; ≥ 98%; Sigma–Aldrich), and the 2,2‐Diphenyl‐1‐picrylhydrazyl (DPPH) assay (95%; Thermo Fisher) as described in Engelhardt et al. (2021) using a high‐throughput method in 96‐well plates (Synergy HTX Multi‐Mode Microplate Reader, BioTek Instruments). For the TEAC and DPPH assays, Trolox standards were used for calibration. Absorbance was measured at 734 nm for TEAC and at 515 nm for DPPH. For total phenolic content (TPC), gallic acid served as the standard, with absorbance measured at 736 nm. All analyses were conducted in duplicates.

### 2.7 Statistical Analysis

Morphological and biochemical data were analyzed separately for each growing season using two‐way ANOVA with drought and salinity as fixed factors. Interactions between factors were also tested. All analytical procedures were performed in duplicates, and the results are presented as mean ± SD. Post hoc comparisons were conducted using Tukey's HSD (*p* ≤ 0.05). Statistical analyses and correlation analyses were performed using the R software (R Core Team 2025). Analyses were conducted separately for each year due to differing numbers of replicates and environmental conditions, as well as to control for year‐to‐year variability. Results tables include standard deviations to represent data variability.

## 3 Results

### 3.1 Morphological Characteristics

In 2023, plant height, stem diameter, fruit number, yield, and leaf number were lower than in 2024, whereas fruit weight was higher (Table 1). Drought‐salinity interactions significantly influenced yield in both years. The highest yield in 2023 occurred under well‐watered conditions (25% of soil weight), whereas in 2024 it was under moderate salinity (0.5% NaCl), which also produced the most fruits. Drought consistently reduced plant height and leaf number in both years, and stem diameter in 2023. Well‐watered tomato plants showed significantly greater height, leaf number, stem diameter, and fruit number than drought‐stressed plants. In 2023, salinity affected plant height, stem diameter, and fruit weight, whereas in 2024, it only impacted stem diameter. The highest values for these attributes were recorded in non‐saline conditions.

**TABLE 1:** The effects of tomato plant morphology in response to varying levels of drought, salinity, and their combination in 2023 and 2024.

	Drought (D)	Salinity (S)	Plant height (cm)	Stem diameter (mm)	Fruit number (per plant)	Yield (gram plant^−1^)	Leaf number (per plant)	Fruit weight (gram fruit^−1^)
2023	25	0	136.9 ± 11.0	10.0 ± 1.8	77 ± 8	1100 ± 122 **a**	70 ± 7	14.5 ± 0.9
		0.5	127.7 ± 8.5	9.1 ± 1.3	75 ± 11	978 ± 108 **ab**	69 ± 4	14.2 ± 0.5
		1	124.5 ± 14.0	9.0 ± 1.5	68 ± 7	918 ± 121 **bc**	66 ± 5	12.2 ± 0.8
	12.5	0	115.1 ± 14.1	8.6 ± 1.2	58 ± 10	826 ± 122 **bcd**	62 ± 9	14.2 ± 0.8
		0.5	103.9 ± 16.6	8.1 ± 1.2	66 ± 9	879 ± 101 **bc**	58 ± 7	13.4 ± 0.8
		1	99.5 ± 17.7	7.5 ± 0.6	57 ± 12	687 ± 155 **d**	55 ± 9	12.0 ± 0.5
	6.25	0	108.8 ± 17.6	8.5 ± 1.0	58 ± 7	792 ± 172 **cd**	59 ± 8	14.0 ± 2.0
		0.5	99.3 ± 15.9	8.4 ± 1.1	66 ± 12	915 ± 193 **bc**	63 ± 9	14.3 ± 1.2
		1	101.9 ± 14.9	8.2 ± 1.6	64 ± 14	799 ± 131 **cd**	59 ± 6	12.2 ± 1.3
	Main effect							
	D	25	129.7 ± 6.4 **a**	9.3 ± 0.6 **a**	74 ± 5 **a**	999 ± 93	68 ± 2 **a**	13.6 ± 1.3
		12.5	106.2 ± 8.0 **b**	8.1 ± 0.6 **b**	61 ± 5 **b**	797 ± 99	58 ± 4 **b**	13.2 ± 1.1
		6.25	103.3 ± 4.9 **b**	8.4 ± 0.2 **b**	63 ± 4 **b**	835 ± 69	60 ± 2 **b**	13.5 ± 1.1
	S	0	120.3 ± 14.7 **a**	9.0 ± 0.8 **a**	64 ± 11	906 ± 169	64 ± 6	14.2 ± 0.3 **a**
		0.5	110.3 ± 15.2 **b**	8.6 ± 0.5 **ab**	69 ± 5	925 ± 50	63 ± 6	13.9 ± 0.5 **a**
		1	108.6 ± 13.8 **b**	8.2 ± 0.7 **b**	63 ± 5	801 ± 116	60 ± 6	12.1 ± 0.1 **b**
	D		***	***	***	***	***	ns
	S		**	*	ns	***	ns	***
	D × S		ns	ns	ns	*	ns	ns
2024	25	0	145.4 ± 27.2	10.4 ± 1.3	103 ± 19 **bc**	1206 ± 218 **bc**	74 ± 6	11.8 ± 0.3
		0.5	153.9 ± 11.4	9.8 ± 0.9	144 ± 14 **a**	1616 ± 149 **a**	76 ± 7	11.5 ± 0.1
		1	140.6 ± 15.2	9.7 ± 1.3	114 ± 14 **b**	1304 ± 157 **b**	76 ± 6	11.2 ± 0.2
	12.5	0	128.5 ± 23.3	11.1 ± 1.5	91 ± 18 **c**	1058 ± 204 **cd**	71 ± 6	11.7 ± 0.1
		0.5	136.6 ± 8.13	9.7 ± 0.9	117 ± 12 **b**	1350 ± 150 **b**	72 ± 7	11.5 ± 0.4
		1	126.0 ± 20.1	9.6 ± 1.0	110 ± 16 **b**	1258 ± 173 **b**	71 ± 7	11.5 ± 0.1
	6.25	0	117.7 ± 21.9	10.9 ± 2.0	73 ± 13 **d**	832 ± 149 **e**	71 ± 6	11.5 ± 0.1
		0.5	122.5 ± 18.5	10.5 ± 1.1	92 ± 16 **c**	1079 ± 182 **cd**	72 ± 6	11.8 ± 0.3
		1	123.5 ± 20.5	10.1 ± 1.7	90 ± 16 **c**	1027 ± 188 **d**	70 ± 6	11.3 ± 0.4
	Main effect							
	D	25	146.6 ± 6.7 **a**	10.0 ± 0.4	120 ± 6	1375 ± 214	75 ± 1 **a**	11.5 ± 0.3
		12.5	130.3 ± 5.6 **b**	10.1 ± 0.8	106 ± 13	1222 ± 149	72 ± 1 **b**	11.6 ± 0.1
		6.25	121.2 ± 3.1 **c**	10.5 ± 0.4	85 ± 10	979 ± 130	71 ± 1 **b**	11.4 ± 0.3
	S	0	130.5 ± 14.0	10.8 ± 0.4 **a**	89 ± 15	1032 ± 188	72 ± 2	11.5 ± 0.3
		0.5	137.6 ± 15.7	10.0 ± 0.4 **b**	118 ± 14	1348 ± 269	74 ± 2	11.6 ± 0.2
		1	130.0 ± 9.2	9.8 ± 0.3 **b**	105 ± 13	1196 ± 148	72 ± 3	11.4 ± 0.2
	D		***	ns	***	***	***	ns
	S		ns	***	***	***	ns	ns
	D × S		ns	ns	***	*	ns	ns

*Note:* Data are expressed as mean ± SD (*n* = 4). Two‐way ANOVA significance: *p ≤ 0.05, **p ≤ 0.01, ***p ≤ 0.001; and ns, not significant difference. If the ANOVA did not reveal any interactions, the main effects of drought or salinity were considered if they were significant. Different lowercase letters indicate significant differences among treatments (*p* ≤ 0.05) according to Tukey's HSD test.

### 3.2 Mineral Content

In 2023, concentrations of phosphorus (P), potassium (K), and sulfur (S) were higher than in 2024, whereas magnesium (Mg) was lower. Calcium (Ca) remained stable across years, and micronutrients (Zn, Mn, Cu, Fe) concentrations showed minimal variation. Sodium (Na) concentration was notably lower in 2023 (Table 2). Drought–salinity interactions significantly affected mineral concentrations, with response patterns differing by year. In 2023, P, Mg, Ca, S, and Mn concentrations were significantly influenced by the combined stress, while in 2024, K and Fe were more responsive. P and Mn concentrations peaked under moderate drought (12.5% of soil weight), while severe drought (6.25% of soil weight) increased Mg and S. The combination of severe drought with moderate salinity (6.25% of soil weight and 0.5% NaCl) elevated Ca and Fe concentrations compared to single stresses. Drought alone affected Zn and Fe concentrations in 2023; well‐watered plants (25% of soil weight) had more Zn, whereas moderate drought increased Fe. In 2024, drought levels raised P and Na concentrations, both of which peaked under severe drought conditions. Na concentration rose inconsistently with salinity in 2024, with higher concentration observed under 0.5% NaCl than under 1.0% NaCl in some treatments. Salinity significantly influenced Zn concentration in 2023 and S, Cu, and Na in 2024. Cu and Na concentrations peaked at 0.5% NaCl in 2024, while S and Zn showed more sensitivity under non‐saline conditions.

**TABLE 2:** The mineral composition of tomato fruit subjected to various treatments of drought, salinity, and their combinations in 2023 and 2024.

	Drought (D)	Salinity (S)	P (mg g^−1^ DM)	K (mg g^−1^ DM)	Mg (mg g^−1^ DM)	Ca (mg g^−1^ DM)	S (mg g^−1^ DM)	Zn (mg g^−1^ DM)	Mn (mg g^−1^ DM)	Cu (mg g^−1^ DM)	Na (mg g^−1^ DM)	Fe (mg g^−1^ DM)
2023	25	0	4.4 ± 1.1 **ab**	24.9 ± 0.9	0.9 ± 0.1 **ab**	1.0 ± 0.2 **a**	2.2 ± 1.1 **abc**	0.06 ± 0.01	0.01 ± 0.00 **abc**	0.10 ± 0.08	0.16 ± 0.10	0.04 ± 0.01
		0.5	4.2 ± 1.0 **ab**	25.7 ± 1.0	1.0 ± 0.1 **ab**	0.9 ± 0.3 **ab**	1.5 ± 0.1 **c**	0.05 ± 0.01	0.01 ± 0.00 **bc**	0.10 ± 0.04	0.53 ± 0.23	0.04 ± 0.01
		1	3.8 ± 0.2 **b**	25.3 ± 0.9	0.9 ± 0.2 **ab**	0.8 ± 0.1 **ab**	2.0 ± 1.0 **abc**	0.07 ± 0.03	0.01 ± 0.00 **abc**	0.08 ± 0.04	0.80 ± 0.35	0.05 ± 0.01
	12.5	0	5.1 ± 1.0 **a**	24.9 ± 1.2	0.8 ± 0.2 **ab**	1.0 ± 0.1 **ab**	1.6 ± 0.1 **bc**	0.06 ± 0.02	0.01 ± 0.00 **a**	0.11 ± 0.07	0.12 ± 0.11	0.05 ± 0.02
		0.5	4.7 ± 0.9 **ab**	25.4 ± 0.6	0.8 ± 0.1 **ab**	0.8 ± 0.1 **ab**	2.5 ± 1.0 **a**	0.06 ± 0.01	0.01 ± 0.00 **abc**	0.08 ± 0.05	0.26 ± 0.15	0.04 ± 0.02
		1	3.6 ± 0.1 **ab**	25.3 ± 0.7	0.9 ± 0.0 **ab**	0.9 ± 0.1 **ab**	1.6 ± 0.1 **c**	0.07 ± 0.01	0.01 ± 0.00 **ab**	0.09 ± 0.04	0.41 ± 0.06	0.06 ± 0.04
	6.25	0	4.0 ± 0.3 **ab**	24.9 ± 0.8	1.0 ± 0.1 **a**	0.8 ± 0.1 **b**	2.7 ± 1.1 **a**	0.05 ± 0.01	0.01 ± 0.00 **c**	0.08 ± 0.02	0.34 ± 0.15	0.04 ± 0.01
		0.5	4.0 ± 0.8 **ab**	24.6 ± 1.8	0.9 ± 0.2 **ab**	1.1 ± 0.7 **a**	1.9 ± 0.8 **abc**	0.04 ± 0.00	0.01 ± 0.00 **abc**	0.09 ± 0.07	0.46 ± 0.21	0.04 ± 0.01
		1	4.5 ± 0.5 **ab**	25.2 ± 0.8	0.8 ± 0.1 **b**	0.7 ± 0.1 **b**	2.4 ± 1.0 **ab**	0.05 ± 0.01	0.01 ± 0.00 **abc**	0.10 ± 0.06	1.26 ± 0.26	0.03 ± 0.01
	Main effect											
	D	25	4.1 ± 0.4	25.3 ± 0.4	0.9 ± 0.1	0.9 ± 0.1	1.9 ± 0.4	0.06 ± 0.01 **a**	0.01 ± 0.00	0.09 ± 0.01	0.50 ± 0.32	0.04 ± 0.01 **ab**
		12.5	4.5 ± 0.7	25.2 ± 0.3	0.9 ± 0.1	0.9 ± 0.1	1.9 ± 0.5	0.05 ± 0.01 **ab**	0.01 ± 0.00	0.09 ± 0.02	0.26 ± 0.15	0.05 ± 0.01 **a**
		6.25	4.2 ± 0.3	24.9 ± 0.3	0.9 ± 0.1	0.9 ± 0.2	2.3 ± 0.4	0.05 ± 0.01 **b**	0.01 ± 0.00	0.09 ± 0.01	0.69 ± 0.50	0.04 ± 0.01 **b**
	S	0	4.5 ± 0.6	24.9 ± 0.0	0.9 ± 0.1	0.9 ± 0.1	2.2 ± 0.6	0.06 ± 0.01 **a**	0.01 ± 0.00	0.09 ± 0.02	0.21 ± 0.12	0.04 ± 0.01
		0.5	4.3 ± 0.4	25.2 ± 0.6	0.9 ± 0.1	0.9 ± 0.2	2.0 ± 0.5	0.05 ± 0.01 **b**	0.01 ± 0.00	0.09 ± 0.01	0.42 ± 0.14	0.04 ± 0.00
		1	4.0 ± 0.5	25.3 ± 0.1	0.9 ± 0.1	0.8 ± 0.1	2.0 ± 0.4	0.06 ± 0.01 **a**	0.01 ± 0.00	0.09 ± 0.01	0.82 ± 0.43	0.05 ± 0.02
	D		ns	ns	ns	ns	ns	**	**	ns	ns	*
	S		ns	ns	ns	ns	ns	*	ns	ns	ns	ns
	D × S		*	ns	**	*	*	ns	**	ns	ns	ns
2024	25	0	3.3 ± 0.1	22.2 ± 0.6 **d**	1.1 ± 0.0	0.9 ± 0.1	1.6 ± 0.0	0.06 ± 0.02	0.01 ± 0.00	0.08 ± 0.03	0.60 ± 0.32	0.05 ± 0.01 **abc**
		0.5	3.5 ± 0.1	23.1 ± 1.0 **cd**	1.1 ± 0.0	0.9 ± 0.1	1.5 ± 0.1	0.06 ± 0.02	0.01 ± 0.00	0.13 ± 0.06	0.94 ± 0.18	0.04 ± 0.01 **bc**
		1	3.4 ± 0.2	22.7 ± 0.5 **cd**	1.1 ± 0.1	1.1 ± 0.2	1.6 ± 0.1	0.06 ± 0.02	0.01 ± 0.00	0.12 ± 0.09	0.90 ± 0.15	0.06 ± 0.02 **abc**
	12.5	0	3.5 ± 0.2	23.3 ± 0.8 **bcd**	1.1 ± 1.1	1.0 ± 0.1	1.7 ± 0.1	0.07 ± 0.03	0.01 ± 0.00	0.12 ± 0.07	0.77 ± 0.23	0.05 ± 0.02 **abc**
		0.5	3.7 ± 0.1	24.6 ± 0.9 **ab**	1.1 ± 0.0	0.9 ± 0.1	1.6 ± 0.1	0.04 ± 0.00	0.01 ± 0.00	0.17 ± 0.10	1.02 ± 0.51	0.04 ± 0.01 **bc**
		1	3.5 ± 0.1	23.4 ± 0.8 **bcd**	1.1 ± 0.0	1.0 ± 0.1	1.6 ± 0.1	0.07 ± 0.03	0.01 ± 0.00	0.17 ± 0.06	0.88 ± 0.18	0.06 ± 0.02 **ab**
	6.25	0	3.5 ± 0.1	23.0 ± 1.0 **cd**	1.1 ± 0.1	1.3 ± 0.7	1.7 ± 0.3	0.05 ± 0.02	0.01 ± 0.00	0.08 ± 0.09	0.90 ± 0.18	0.05 ± 0.01 **bc**
		0.5	3.5 ± 0.2	23.9 ± 0.8 **abc**	1.1 ± 0.0	1.0 ± 0.1	1.5 ± 0.1	0.06 ± 0.02	0.01 ± 0.00	0.18 ± 0.10	1.22 ± 0.33	0.07 ± 0.03 **a**
		1	3.5 ± 0.2	24.7 ± 0.5 **a**	1.1 ± 0.0	0.8 ± 0.1	1.5 ± 0.1	0.06 ± 0.02	0.01 ± 0.00	0.21 ± 0.13	1.00 ± 0.17	0.04 ± 0.01 **c**
	Main effect											
	D	25	3.4 ± 0.1 **b**	22.7 ± 0.5	1.1 ± 0.0	1.0 ± 0.1	1.5 ± 0.1	0.06 ± 0.00	0.01 ± 0.00	0.11 ± 0.03	0.81 ± 0.19 **b**	0.05 ± 0.01
		12.5	3.6 ± 0.1 **a**	23.8 ± 0.8	1.1 ± 0.0	1.0 ± 0.1	1.6 ± 0.1	0.06 ± 0.02	0.01 ± 0.00	0.15 ± 0.03	0.89 ± 0.13 **ab**	0.05 ± 0.01
		6.25	3.5 ± 0.0 **a**	23.9 ± 0.9	1.1 ± 0.0	1.0 ± 0.3	1.6 ± 0.1	0.06 ± 0.01	0.01 ± 0.00	0.15 ± 0.07	1.04 ± 0.16 **a**	0.05 ± 0.02
	S	0	3.4 ± 0.1	22.8 ± 0.4	1.1 ± 0.0	1.1 ± 0.2	1.6 ± 0.1 **a**	0.06 ± 0.01	0.01 ± 0.00	0.09 ± 0.02 **b**	0.76 ± 0.15 **b**	0.05 ± 0.00
		0.5	3.5 ± 0.1	23.8 ± 0.8	1.1 ± 0.0	1.0 ± 0.1	1.5 ± 0.1 **b**	0.05 ± 0.01	0.01 ± 0.00	0.16 ± 0.03 **a**	1.06 ± 0.14 **a**	0.05 ± 0.02
		1	3.4 ± 0.1	23.6 ± 1.0	1.1 ± 0.0	1.0 ± 0.2	1.5 ± 0.1 **b**	0.06 ± 0.01	0.01 ± 0.00	0.17 ± 0.05 **a**	0.93 ± 0.06 **ab**	0.05 ± 0.01
	D		***	***	ns	ns	ns	ns	ns	ns	*	ns
	S		ns	***	ns	ns	**	ns	ns	**	**	ns
	D × S		ns	**	ns	ns	ns	ns	ns	ns	ns	**

*Note:* Data are expressed as mean ± SD (*n* = 4). Two‐way ANOVA significance: *p ≤ 0.05, **p ≤ 0.01, ***p ≤ 0.001; and ns, not significant difference. If the ANOVA did not reveal any interactions, the main effects of drought or salinity were considered if they were significant. Different lowercase letters indicate significant differences among treatments (*p* ≤ 0.05) according to Tukey's HSD test.

### 3.3 Carotenoids

Carotenoid concentrations were generally higher in 2024 than in 2023 (Table 3). Drought–salinity interactions significantly influenced *β*‐carotene concentrations in both years. The highest *β*‐carotene concentrations were recorded in 2023 at 1.0% NaCl, corresponding to 25% of the soil weight, and in 2024 at 0.5% NaCl, corresponding to 12.5% of the soil weight. Lycopene and lutein were only significantly affected in 2024. Lycopene and lutein in 2024 demonstrated the highest percentages, at 25% and 6.25% of the soil weight, respectively, under non‐saline conditions. The lowest concentration of *β*‐carotene was observed under combined stress: 6.25% of soil weight with 0.5% NaCl in 2023 and 12.5% of soil weight with 1.0% NaCl in 2024. In 2024, lycopene reached its lowest concentration at 6.25% of soil weight with 1.0% NaCl, while lutein was lowest under moderate drought (12.5% of soil weight) and moderate salinity (0.5% NaCl).

**TABLE 3:** Drought, salinity, and their combination effects on carotenoid concentrations in tomato fruit in 2023 and 2024. Data are expressed as mean ± SD (*n* = 4).

	Drought (D)	Salinity (S)	Lycopene (μg g^−1^ DM)	*β*‐carotene (μg g^−1^ DM)	Lutein (μg g^−1^ DM)
2023	25	0	172.1 ± 20.9	31.6 ± 3.6 **ab**	9.9 ± 1.1
		0.5	157.5 ± 24.5	29.3 ± 2.8 **abc**	10.2 ± 0.6
		1	170.8 ± 28.8	32.8 ± 3.0 **a**	10.5 ± 1.0
	12.5	0	178.0 ± 17.6	28.7 ± 1.6 **bc**	10.0 ± 0.6
		0.5	180.1 ± 17.5	31.5 ± 2.2 **ab**	10.0 ± 0.4
		1	162.0 ± 17.3	30.5 ± 1.3 **abc**	9.3 ± 0.5
	6.25	0	190.5 ± 20.3	29.6 ± 2.1 **abc**	10.2 ± 0.8
		0.5	176.3 ± 25.2	27.3 ± 2.4 **c**	10.3 ± 0.9
		1	166.1 ± 14.0	30.3 ± 2.0 **abc**	10.0 ± 0.9
	Main effect				
	D	25	166.8 ± 8.1	31.2 ± 1.8	10.2 ± 0.3
		12.5	173.3 ± 9.9	30.2 ± 1.4	9.8 ± 0.4
		6.25	177.7 ± 12.3	29.0 ± 1.5	10.2 ± 0.2
	S	0	180.2 ± 9.4	30.0 ± 1.4	10.0 ± 0.2
		0.5	171.3 ± 12.2	29.3 ± 2.1	10.2 ± 0.2
		1	166.3 ± 4.4	31.2 ± 1.4	9.9 ± 0.6
	D		ns	*	ns
	S		ns	*	ns
	D × S		ns	*	ns
2024	25	0	361.5 ± 28.1 **a**	50.0 ± 6.4 **ab**	14.1 ± 0.9 **bc**
		0.5	320.1 ± 49.6 **abc**	44.2 ± 4.2 **bcd**	13.3 ± 0.9 **c**
		1	346.4 ± 25.9 **ab**	44.9 ± 3.5 **abcd**	15.7 ± 0.8 **ab**
	12.5	0	304.2 ± 38.9 **abc**	44.0 ± 0.7 **cd**	13.5 ± 1.1 **c**
		0.5	319.2 ± 32.9 **abc**	50.9 ± 3.4 **a**	15.5 ± 1.7 **ab**
		1	294.7 ± 38.8 **bc**	42.8 ± 3.0 **d**	14.3 ± 1.2 **bc**
	6.25	0	342.4 ± 31.5 **ab**	48.5 ± 3.4 **abcd**	16.5 ± 1.3 **a**
		0.5	293.8 ± 28.7 **bc**	44.4 ± 4.5 **bcd**	15.5 ± 0.7 **ab**
		1	278.9 ± 58.3 **c**	49.1 ± 1.5 **abc**	14.4 ± 0.8 **bc**
	Main effect				
	D	25	342.7 ± 21.0	46.4 ± 3.2	14.3 ± 1.2
		12.5	306.0 ± 12.4	45.9 ± 4.4	14.4 ± 1.0
		6.25	305.0 ± 33.2	47.3 ± 2.6	15.5 ± 1.1
	S	0	336.1 ± 29.2	47.5 ± 3.1	14.7 ± 1.6
		0.5	311.0 ± 14.9	46.5 ± 3.8	14.8 ± 1.3
		1	306.7 ± 35.3	45.6 ± 3.2	14.8 ± 0.8
	D		**	ns	**
	S		*	ns	ns
	D × S		*	***	***

*Note:* Two‐way ANOVA significance: *p ≤ 0.05, **p ≤ 0.01, ***p ≤ 0.001; and ns, not significant difference. If the ANOVA did not reveal any interactions, the main effects of drought or salinity were considered if they were significant. Different lowercase letters indicate significant differences among treatments (*p* ≤ 0.05) according to Tukey's HSD test.

### 3.4 Phenolic Compounds

Concentrations of all phenolic compounds increased in 2024 compared to 2023 (Table 4). Drought–salinity interactions significantly affected chlorogenic acid concentrations in 2023 and naringenin chalcone concentrations in both 2023 and 2024. In 2023, both compounds reached their peak under severe salinity (1.0% NaCl) with well‐watered conditions (25% of the soil's weight). The lowest concentrations occurred under moderate drought combined with severe salinity (12.5% of soil weight and 1.0% NaCl). In 2024, naringenin chalcone increased under severe drought (6.25% of soil weight) without salinity. Chlorogenic acid concentration also increased under severe drought, but was reduced by salinity compared to the control.

**TABLE 4:** Impact of varying levels of drought, salinity, and their combination on the chlorogenic acid, quercetin‐3‐rutinoside, and naringenin chalcone content in tomato fruits during 2023 and 2024.

	Drought (D)	Salinity (S)	Chlorogenic acid (mg g^−1^ DM)	Quercetin‐3‐rutinoside (mg g^−1^ DM)	Naringenin chalcone (mg g^−1^ DM)
2023	25	0	0.27 ± 0.04 **bc**	0.31 ± 0.05	0.05 ± 0.00 **ab**
		0.5	0.28 ± 0.06 **bc**	0.36 ± 0.08	0.05 ± 0.01 **b**
		1	0.37 ± 0.08 **a**	0.37 ± 0.07	0.07 ± 0.01 **a**
	12.5	0	0.32 ± 0.02 **abc**	0.35 ± 0.03	0.06 ± 0.01 **ab**
		0.5	0.29 ± 0.06 **bc**	0.35 ± 0.03	0.05 ± 0.01 **ab**
		1	0.24 ± 0.04 **c**	0.32 ± 0.04	0.05 ± 0.00 **b**
	6.25	0	0.32 ± 0.05 **abc**	0.35 ± 0.04	0.06 ± 0.01 **ab**
		0.5	0.30 ± 0.05 **abc**	0.35 ± 0.10	0.06 ± 0.02 **ab**
		1	0.33 ± 0.03 **ab**	0.32 ± 0.05	0.06 ± 0.02 **ab**
	Main effect				
	D	25	0.31 ± 0.06	0.35 ± 0.03	0.06 ± 0.01
		12.5	0.28 ± 0.04	0.34 ± 0.02	0.05 ± 0.01
		6.25	0.32 ± 0.02	0.34 ± 0.02	0.06 ± 0.00
	S	0	0.30 ± 0.03	0.34 ± 0.02	0.06 ± 0.01
		0.5	0.29 ± 0.01	0.36 ± 0.01	0.05 ± 0.01
		1	0.32 ± 0.07	0.34 ± 0.03	0.06 ± 0.01
	D		*	ns	ns
	S		ns	ns	ns
	D × S		***	ns	**
2024	25	0	0.47 ± 0.04	0.56 ± 0.07	0.09 ± 0.01 **b**
		0.5	0.45 ± 0.04	0.57 ± 0.05	0.07 ± 0.02 **b**
		1	0.47 ± 0.14	0.62 ± 0.09	0.08 ± 0.01 **b**
	12.5	0	0.45 ± 0.01	0.54 ± 0.07	0.09 ± 0.01 **b**
		0.5	0.44 ± 0.02	0.56 ± 0.06	0.06 ± 0.01 **b**
		1	0.41 ± 0.09	0.56 ± 0.03	0.06 ± 0.02 **b**
	6.25	0	0.64 ± 0.19	0.61 ± 0.07	0.15 ± 0.04 **a**
		0.5	0.47 ± 0.08	0.55 ± 0.07	0.08 ± 0.01 **b**
		1	0.49 ± 0.14	0.53 ± 0.09	0.09 ± 0.04 **b**
	Main effect				
	D	25	0.46 ± 0.01 **b**	0.58 ± 0.03	0.08 ± 0.01
		12.5	0.43 ± 0.02 **b**	0.55 ± 0.01	0.07 ± 0.02
		6.25	0.53 ± 0.09 **a**	0.56 ± 0.04	0.10 ± 0.04
	S	0	0.52 ± 0.10 **a**	0.57 ± 0.04	0.11 ± 0.03
		0.5	0.45 ± 0.02 **b**	0.56 ± 0.01	0.07 ± 0.01
		1	0.45 ± 0.04 **b**	0.57 ± 0.05	0.08 ± 0.02
	D		**	ns	***
	S		*	ns	***
	D × S		ns	ns	**

*Note:* Data are expressed as mean ± SD (*n* = 4). Two‐way ANOVA significance: *p ≤ 0.05, **p ≤ 0.01, ***p ≤ 0.001; and ns, not significant difference. If the ANOVA did not reveal any interactions, the main effects of drought or salinity were considered if they were significant. Different lowercase letters indicate significant differences among treatments (*p* ≤ 0.05) according to Tukey's HSD test.

### 3.5 Antioxidant Activity Measured by TEAC, DPPH, and TPC Assays

Antioxidant activities measured by TEAC and DPPH were higher in 2024 than in 2023, while total phenolic content (TPC) was lower (Table 5). Significant drought–salinity interactions were observed for TEAC and DPPH in 2023, but not for TPC. The antioxidant activity (TEAC) was elevated under moderate and severe combined stress (12.5% of soil weight and 0.5% NaCl; 6.25% of soil weight and 1.0% NaCl) in both years. In 2023, antioxidant activity (DPPH) peaked under severe drought and salinity conditions (6.25% of soil weight and 1.0% NaCl), comparable to levels under moderate drought (12.5% of soil weight) in non‐saline conditions. In 2024, antioxidant activity (DPPH) increased under 1.0% NaCl and was comparable to non‐saline conditions in well‐watered environments. TPC in 2024 increased under moderate drought conditions, combined with 1.0% NaCl. The lowest antioxidant activity (TPC) values were observed under well‐watered, non‐saline (25% of soil weight) and moderate drought conditions (12.5% of soil weight).

**TABLE 5:** Antioxidant activity in tomato plants grown under drought, salinity, and their combination in 2023 and 2024.

	Drought (D)	Salinity (S)	TEAC (mmol TE g^−1^ DM)	DPPH (mmol TE g^−1^ DM)	TPC (mg GA g^−1^ DM)
2023	25	0	0.022 ± 0.022 **abc**	0.013 ± 0.000 **cde**	2.8 ± 0.5
		0.5	0.020 ± 0.003 **c**	0.011 ± 0.002 **de**	2.8 ± 0.5
		1	0.022 ± 0.002 **bc**	0.014 ± 0.001 **bcd**	2.8 ± 0.5
	12.5	0	0.021 ± 0.001 **c**	0.011 ± 0.001 **e**	2.8 ± 0.6
		0.5	0.026 ± 0.002 **a**	0.016 ± 0.002 **ab**	2.6 ± 0.1
		1	0.024 ± 0.003 **ab**	0.015 ± 0.003 **abc**	2.5 ± 0.2
	6.25	0	0.021 ± 0.003 **c**	0.013 ± 0.001 **cde**	2.9 ± 0.4
		0.5	0.021 ± 0.003 **bc**	0.012 ± 0.001 **de**	3.0 ± 0.6
		1	0.025 ± 0.001 **a**	0.017 ± 0.002 **a**	2.5 ± 0.1
	Main effect				
	D	25	0.021 ± 0.001	0.013 ± 0.001	2.8 ± 0.0
		12.5	0.023 ± 0.003	0.014 ± 0.003	2.6 ± 0.2
		6.25	0.022 ± 0.002	0.014 ± 0.003	2.8 ± 0.3
	S	0	0.021 ± 0.001	0.012 ± 0.001	2.8 ± 0.1
		0.5	0.022 ± 0.003	0.013 ± 0.002	2.8 ± 0.2
		1	0.024 ± 0.002	0.015 ± 0.002	2.6 ± 0.2
	D		**	*	ns
	S		***	***	ns
	D × S		***	***	ns
2024	25	0	0.027 ± 0.002 **d**	0.019 ± 0.001 **e**	1.0 ± 0.1 **d**
		0.5	0.031 ± 0.002 **abc**	0.028 ± 0.002 **ab**	1.6 ± 0.1 **c**
		1	0.032 ± 0.002 **abc**	0.029 ± 0.001 **a**	1.6 ± 0.3 **c**
	12.5	0	0.026 ± 0.002 **d**	0.019 ± 0.001 **de**	1.0 ± 0.2 **d**
		0.5	0.034 ± 0.002 **a**	0.023 ± 0.002 **c**	2.4 ± 0.7 **b**
		1	0.033 ± 0.003 **ab**	0.023 ± 0.002 **cd**	3.1 ± 0.5 **a**
	6.25	0	0.029 ± 0.004 **cd**	0.024 ± 0.007 **bc**	1.2 ± 0.4 **cd**
		0.5	0.030 ± 0.003 **bcd**	0.022 ± 0.001 **cde**	1.7 ± 0.3 **c**
		1	0.034 ± 0.001 **a**	0.022 ± 0.002 **cde**	1.7 ± 0.1 **c**
	Main effect				
	D	25	0.030 ± 0.003	0.025 ± 0.006	1.4 ± 0.3
		12.5	0.031 ± 0.004	0.021 ± 0.002	2.2 ± 1.1
		6.25	0.031 ± 0.003	0.022 ± 0.001	1.6 ± 0.3
	S	0	0.027 ± 0.002	0.020 ± 0.003	1.1 ± 0.1
		0.5	0.032 ± 0.002	0.024 ± 0.003	1.9 ± 0.4
		1	0.033 ± 0.001	0.025 ± 0.004	2.2 ± 0.8
	D		ns	***	***
	S		***	***	***
	D × S		**	***	***

*Note:* Data are expressed as mean ± SD (*n* = 4). Two‐way ANOVA significance: *p ≤ 0.05, **p ≤ 0.01, ***p ≤ 0.001; and ns, not significant difference. If the ANOVA did not reveal any interactions, the main effects of drought or salinity were considered if they were significant. Different lowercase letters indicate significant differences among treatments (*p* ≤ 0.05) according to Tukey's HSD test.

### 3.6 Correlation Analysis

**FIGURE 2:**
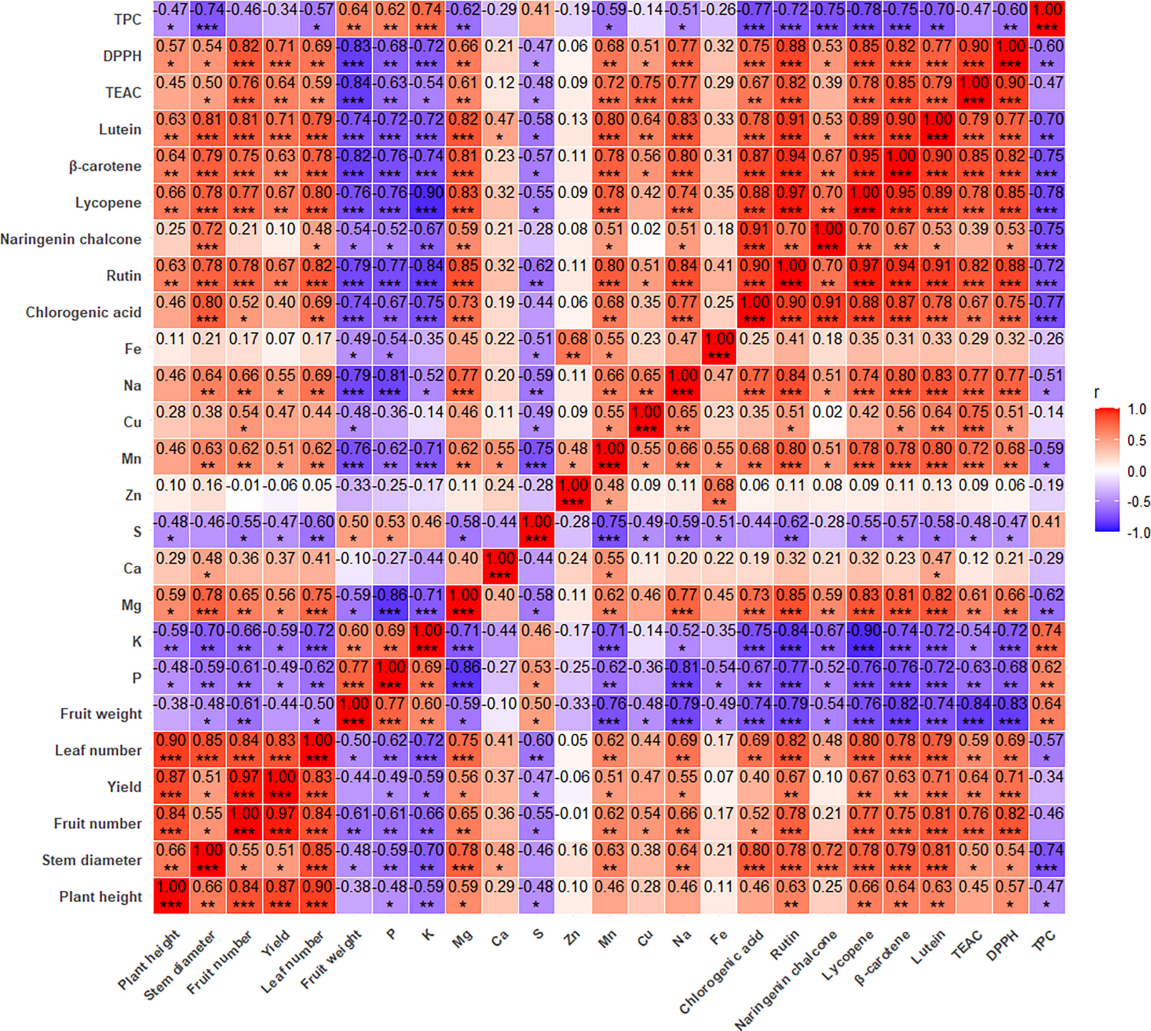
Pearson's correlation analysis of morphological parameters, minerals, and secondary metabolites in tomato fruit, grown over two consecutive years (2023 and 2024), was conducted under various levels of drought, salinity, and their combination. Asterisks indicate significant correlations at the *p ≤ 0.05, **p ≤ 0.01, ***p ≤ 0.001. Heatmap showing Pearson's correlation coefficients among morphological parameters, mineral elements, carotenoids, phenolic compounds, and antioxidant activities measured in tomato fruits grown under drought, salinity, and combined stress treatments over two consecutive seasons (2023 and 2024). Asterisks indicate statistically significant correlations (*p* ≤ 0.05, *p* ≤ 0.01, and *p* ≤ 0.001).

Significant correlations were observed among morphological traits, mineral composition, and secondary metabolites in tomato fruits (Figure 2). Morphological parameters, including plant height, stem diameter, fruit number, yield, and leaf number, were positively correlated with magnesium (*r* = 0.56–0.78, *p* ≤ 0.05–0.001) and phenolic compounds (*r* = 0.48–0.82, *p* ≤ 0.05–0.001). Positive correlations were also found with carotenoids (*r* = 0.63–0.81, *p* ≤ 0.01–0.001) and antioxidant activities (TEAC and DPPH; *r* = 0.50–0.82, *p* ≤ 0.05–0.001). In contrast, these traits showed negative correlations with phosphorus, potassium, and sulfur (*r* = −0.72 to −0.47, *p* ≤ 0.05–0.001), as well as total phenolic content (TPC) (*r* = −0.74 to −0.47, *p* ≤ 0.05). Magnesium positively correlated with phenolic compounds (*r* = 0.59–0.85, *p* ≤ 0.01–0.001), carotenoids (*r* = 0.81–0.83, *p* ≤ 0.001), and antioxidant activity (TEAC and DPPH; *r* = 0.61–0.66, *p* ≤ 0.05), but showed negative correlations with fruit weight (*r* = −0.59, *p* ≤ 0.05), TPC (*r* = −0.62, *p* ≤ 0.01), and phosphorus, potassium, sulfur (*r* = −0.86 to −0.58, *p* ≤ 0.05–0.001). Phosphorus and potassium were positively correlated with TPC (*r* = 0.62–0.74, *p* ≤ 0.01–0.001), but negatively associated with phenolic compounds, carotenoids, and antioxidant activity (TEAC and DPPH) (*r* = −0.90 to −0.52, *p* ≤ 0.05–0.001). Phenolic compounds positively correlated with carotenoids (*r* = 0.53–0.97, *p* ≤ 0.05–0.001) and antioxidant activity (TEAC and DPPH) (*r* = 0.53–0.88, *p* ≤ 0.05–0.001). Carotenoids also positively correlated with TEAC and DPPH (*r* = 0.77–0.85, *p* ≤ 0.001). The Total Phenolic Content (TPC) exhibited strong negative correlations with carotenoids (*r* = −0.78 to −0.70, *p* ≤ 0.01–0.001) and DPPH (*r* = −0.60, *p* ≤ 0.05).

## 4 Discussion

### 4.1 Morphological Characteristics

Drought and salinity stress reduced tomato yield in both years, with fruit number significantly affected in 2024. Yearly differences may relate to environmental variation. Cooler and less sunny conditions in 2023 likely limited reproductive development, whereas higher temperatures in 2024 intensified stress sensitivity (Figures S1 and S2). Similar seasonal and environmental interactions influencing tomato growth and yield have been reported before (Diouf et al. 2020; Francesca et al. 2022; Roșca et al. 2023). Both drought and salinity disrupt photosynthesis, nutrient uptake, and antioxidant defenses, reducing yield and fruit quality (Yavuz et al. 2023). Their increasing prevalence is associated with global freshwater scarcity (Ahmed et al. 2019). Resi is an aromatic cocktail tomato cultivar adapted to outdoor cultivation and extended harvests. Cocktail tomato cultivars possess beneficial drought‐adaptive traits, such as improved osmotic regulation and antioxidant activity, which enable them to maintain yield and fruit quality under moderate water limitations (Petrović et al. 2021). Our study indicated that drought had a stronger negative impact on yield than salinity, with a yield decrease of approximately 28% under drought and 17% under salinity. A combination of both conditions led to a similar reduction (approximately 27%), suggesting that severe drought may have saturated stress‐response mechanisms (Zandalinas and Mittler 2022). Increasing drought intensity also reduced plant height, stem diameter, fruit and leaf number, likely due to impaired water and nutrient transport. These results align with previous studies that have shown a drought‐induced reduction in tomato growth and productivity (Akhoundnejad 2020; Jangid and Dwivedi 2016; Kazemi et al. 2021; Petrović et al. 2019; Sivakumar and Srividhya 2016; Turan et al. 2023). Salinity stress caused progressive reductions in plant height, stem diameter, and fruit weight, driven by osmotic stress that limits water uptake and ionic stress that disrupts nutrient balance (Carillo et al. 2020; Munns and Tester 2008). Similar growth reductions have been observed in other tomato cultivars (Cuartero & Fernandez‐Munoz, Cuartero and Fernandez‐Munƒoz 1999), reflecting cultivar‐specific differences in tolerance. Plants in well‐watered, non‐saline conditions consistently performed best, confirming that adequate irrigation mitigates salt stress and supports productivity (Karimzadeh et al. 2024).

### 4.2 Mineral Content

Drought–salinity interactions influenced mineral accumulation differently across the two study years; however, overall variations were minor, suggesting that the stress treatments did not critically impair plant survival. Environmental differences, particularly higher temperatures in 2024, may have increased osmotic imbalances and changed ion transport dynamics. Salinity beyond 1.0% NaCl would likely further increase Na^+^ accumulation, intensifying ionic and osmotic stress. Under moderate or severe drought in non‐saline conditions, phosphorus, magnesium, sulfur, and manganese increased. In contrast, calcium and iron rose slightly under combined stress. These mineral adjustments reflect the selective retention and redistribution of ions during drought, helping to preserve cell turgor and maintain metabolic activity (Ahmed et al. 2023; Hasanuzzaman et al. 2018). This aligns with Angon et al. (2022) and Zandalinas and Mittler (2022), who suggest that the combined effects of stress depend on trait, genotype, and environment. Drought increased phosphorus and sodium concentrations in 2024, likely due to reduced biomass and limited leaching rather than increased uptake (He and Dijkstra 2014). Calcium decreased during severe drought and salinity due to competition with Na^+^ at root membranes and soil exchange sites, hindering Ca^2+^ transport to fruits (Atta et al. 2023; Munns and Tester 2008). High Na^+^ concentration disrupts ion balance and lowers essential mineral concentrations, such as K^+^, Ca^2+^, and Mg^2+^. In response, plants increase K^+^ uptake to maintain the K^+^/Na^+^ ratio, stabilizing osmotic potential and enzymatic functions (Bhardwaj et al. 2025; Hasanuzzaman et al. 2018; Parihar et al. 2015). Copper concentration increased under moderate salinity, possibly due to membrane damage enhancing Cu^2+^ uptake (Fal et al. 2022). Zinc and sulfur concentrations were higher in non‐saline conditions, reflecting improved nutrient uptake under lower ionic competition and unrestricted sulfate uptake (Machado and Serralheiro 2017; Shahid et al. 2020). Magnesium plays a central role in stabilizing chlorophyll, maintaining photosynthetic efficiency, and supporting antioxidant defense (Ahmed et al. 2023; Ishfaq et al. 2022; Nas and Kömürkara Zengin 2024). During drought, magnesium concentration can rise, reflecting reduced biomass dilution and an increased need for reactive oxygen species (ROS) detoxification. The connection between Mg^2+^ and antioxidants suggests that Mg^2+^ contributes to redox balance and supports metabolic stability under stress. Overall, these mineral trends indicate an active ionic adjustment in tomatoes to maintain nutrient homeostasis under combined stress.

### 4.3 Carotenoids

Carotenoid accumulation varied in response to drought and salinity across years. In 2023, there were no significant changes in lycopene or lutein concentration, while *β*‐carotene increased moderately under stress. However, in 2024 both lycopene and lutein significantly decreased under combined drought and salinity, whereas *β*‐carotene showed interactions with these conditions. This year‐to‐year inconsistency may result from environmental factors affecting pigment biosynthesis, as higher temperatures and light intensity in 2024 likely enhanced oxidative degradation and altered carotenoid pathways, as previously observed by Helyes et al. (2007) and Slimestad and Verheul (2005). Lycopene peaked under well‐watered conditions, reflecting optimal photosynthesis and reduced oxidative stress. Under drought and salinity, the concentration decreased due to both inhibited synthesis and oxidative degradation (Havaux 2014; Massaretto et al. 2018). Combined stress further intensified this reduction, reflecting lycopene's high sensitivity to oxidative imbalance. *β*‐Carotene concentration increased under severe salinity and moderate combined stress, indicating a protective response against ROS and maintaining membrane integrity. In addition to its antioxidant function, *β*‐carotene acts as a precursor to abscisic acid (ABA), which helps promote stomatal closure and regulate osmotic balance during dehydration (Águila Ruiz‐Sola et al. 2014; Sah et al. 2016). Under severe combined stress, excessive ROS may cleave *β*‐carotene, explaining its decline (Leiva‐Ampuero et al. 2020). Lutein concentration increased during severe drought, highlighting its role in protecting chloroplasts (Nisar et al. 2015). In contrast, its decline under moderate drought and salinity suggests that it is due to biosynthetic inhibition by oxidative and ionic stress. Overall, carotenoid metabolism adjusts to combined oxidative and osmotic stress, with *β*‐carotene contributing to antioxidant protection and ABA‐related stress signaling as lycopene concentration decreases.

### 4.4 Phenolic Compounds

The main phenolic compounds in tomatoes include flavonoids such as rutin, naringenin chalcone, and quercetin glycosides, as well as hydroxycinnamic acids like chlorogenic and caffeic acid (Martí et al. 2018). Phenolic responses to drought and salinity varied by compound and stress intensity across both years. In 2023, chlorogenic acid and naringenin chalcone increased under severe salinity, while in 2024, severe drought further raised chlorogenic acid concentration, particularly at drought level 6.25% of soil weight. These results indicate that drought and salinity trigger similar antioxidant pathways, with varying responses based on environmental conditions. Both stressors increase phenolic compound accumulation via the phenylpropanoid pathway, improving the plant's ability to scavenge reactive oxygen species and enhancing tolerance to oxidative stress (Klunklin and Savage 2017; Kumar et al. 2023; Rao and Zheng 2025). Rutin (quercetin‐3‐rutinoside) remained stable across treatments, confirming its essential role in antioxidant defense and UV protection (Ismail et al. 2016). Increases in fruit phenolics in tomatoes under drought or salinity support their role as indicators of oxidative stress (Botella et al. 2021; Conti et al. 2022; Dere et al. 2022). The variation from year to year indicates that environmental factors, such as temperature and light intensity, influence phenolic accumulation and affect the plant's antioxidant response under stress. Increased chlorogenic acid and naringenin chalcone concentration under severe stress are particularly important in maintaining redox balance and preserving fruit quality under extreme environmental conditions.

### 4.5 Antioxidant Activity Measured by TEAC, DPPH, and TPC Assays

Antioxidant activity measured by the TEAC, DPPH, and TPC assays varied in response to the intensity of drought and salinity. In 2023, TEAC and DPPH showed small but statistically significant increases under moderate salinity and its combination with drought stress, while TPC remained unchanged. These modest enhancements suggest the activation of stress‐responsive mechanisms, even under relatively mild oxidative stress. In 2024, stronger increases were observed across all three assays, particularly under moderate to severe salinity and drought conditions, indicating a greater oxidative challenge in warmer seasonal conditions. Year‐to‐year differences demonstrate the sensitivity of antioxidant activity to environmental changes. This sensitivity is especially noticeable when light and water availability change. Abiotic stress leads to the accumulation of reactive oxygen species, which activate both enzymatic and non‐enzymatic antioxidants, including phenolics and flavonoids, to maintain redox balance (Koffler et al. 2014; Shah and Smith 2020). Increased antioxidant activity has been observed in tomatoes under water deficit, indicating a coordinated activation of phenolic and carotenoid metabolism for the detoxification of reactive oxygen species (Conti et al. 2022; Dere et al. 2022). The simultaneous rise in total phenolic content under combined stress underscores the connection between phenolic metabolism and antioxidant activity, as part of the redox regulation process. Under well‐watered and non‐saline conditions, the generation of ROS remains low, which minimizes the need for antioxidant induction. Overall, drought and salinity enhanced antioxidant defenses in tomatoes, though the extent and statistical significance varied depending on the severity of the stress and the seasonal environment.

### 4.6 Correlation Analysis

Pearson's correlation analysis revealed a significant positive relationship between plant morphology, magnesium content, and secondary metabolites, suggesting that under stress, plants regulate Mg^2+^ uptake to support growth and redox stability. Magnesium is essential for stabilizing chlorophyll, maintaining photosynthetic efficiency, activating antioxidant enzymes, and promoting the synthesis of phenolics and carotenoids during stress (Ahmed et al. 2023; Hamedeh et al. 2022). The relationship between Mg^2+^ and phenolic or carotenoid compounds may not exhibit direct causation, but rather a co‐regulated adjustment, where adequate Mg^2+^ enhances photosynthetic efficiency and antioxidant enzyme activity, thereby supporting redox homeostasis. Under stress, resource allocation appears to favor osmotic regulation and antioxidant defense over biomass accumulation, aligning with the observed negative correlations between nutrient concentrations and fruit weight. Elevated magnesium concentration may also interfere with the uptake of phosphorus, potassium, and sulfur by altering ion balance. Most morphological traits positively correlated with antioxidant activities (TEAC, DPPH), carotenoids, and phenolic compounds, indicating that plants with higher secondary metabolism perform better under stress. However, some negative associations (e.g., with TPC) suggest this relationship varies by compound type and stress intensity. Flavonoids and carotenoids serve as non‐enzymatic scavengers of ROS, mitigating oxidative damage and preserving physiological integrity (Patil et al. 2024; Shah and Smith 2020; Shomali et al. 2022). Conversely, negative correlations emerged between morphology and both sulfur content and total phenolic content (TPC). While sulfur accumulation may boost stress metabolites such as glutathione, it can also inhibit growth under severe stress (Lattanzio 2021; Ma et al. 2020; Zenda et al. 2021). The phosphorus, potassium, and sulfur contents were negatively correlated with morphology and secondary metabolites, reflecting nutrient limitation. Under nutrient‐rich conditions, plants allocate more resources to growth, whereas stress redirects metabolism toward defense and antioxidant production (Li et al. 2021; Sung et al. 2016).

## 5 Conclusions

This study showed that drought had a stronger negative effect on tomato growth and yield than salinity, while combined stress further intensified these effects without a strictly additive interaction. Comparing the two growing seasons underscores how temperature and light conditions significantly affect stress responses. Moderate stress levels slightly increased potassium uptake, maintaining the K^+^/Na^+^ ratio, whereas severe drought and salinity consistently reduced calcium content. Reductions in *β*‐carotene and lycopene, alongside stable lutein concentration, indicate shifts in antioxidant strategies, while increases in chlorogenic acid and naringenin chalcone highlight oxidative defenses. Enhanced antioxidant activities (TEAC, DPPH, TPC) emphasize redox adjustments under stress. The differences in secondary metabolite accumulation between years highlight those environmental influences on biochemical responses, even with the same stress treatments. These findings indicate that tomato adaptation to drought and salinity involves complex interactions between mineral nutrition, secondary metabolism affecting antioxidant activities, and environmental factors. Effective water management is vital for maintaining tomato yield and quality under climatic stress.

## Author Contributions

N.A.P. conceptualized the study, conducted the investigation, performed formal data analysis, curated the data, prepared the visualizations, and wrote the original draft of the manuscript, as well as reviewed and edited subsequent versions. T.P. contributed to the investigation and methodology and provided supervision, critical review, and editing of the manuscript. S.N. contributed to the conceptualization and methodology of the study, provided resources and supervision, and critically reviewed and edited the manuscript. All authors discussed the results and contributed to the writing of the final manuscript.

## Funding

This work was supported by the Division of Quality and Sensory of Plant Products, Department of Crop Science, Georg‐August‐Universität Göttingen, Germany. Niken Ayu Permatasari received a scholarship from the IPB University, Indonesia.

## Supporting information


**Data S1:** Supporting Information.

## Data Availability

The data that support the findings of this study are available from the corresponding author upon reasonable request.
