# Supplementary material for: Impact of Drought, Salinity, and Their Combination on Growth, Mineral Content, and Plant Secondary Metabolites of Tomatoes ( Solanum lycopersicum L.)
Source: Physiol Plant. 2025 Dec 30;178(1):e70725. doi: 10.1111/ppl.70725 (PMC12751611; doi:10.1111/ppl.70725)
Supplement: Supplementary file 1 — Data S1: Supporting Information. [file PPL-178-e70725-s001.docx]

**Supplementary Material**


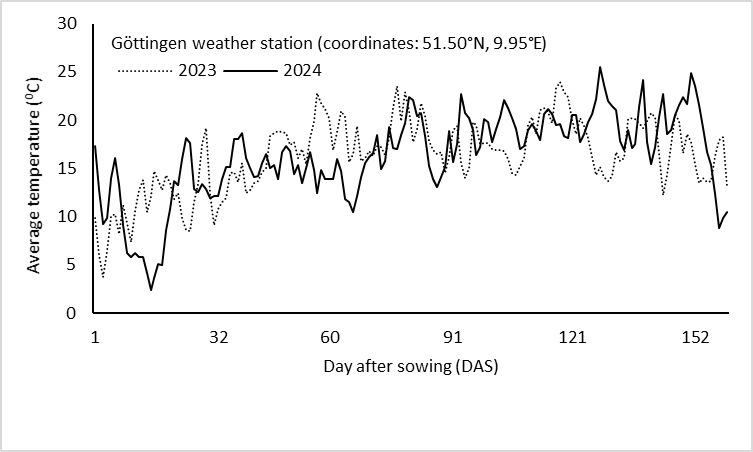


**Supplemental Figure 1**. Average temperature in Göttingen (^0^C) measured during the growing seasons 2023 and 2024


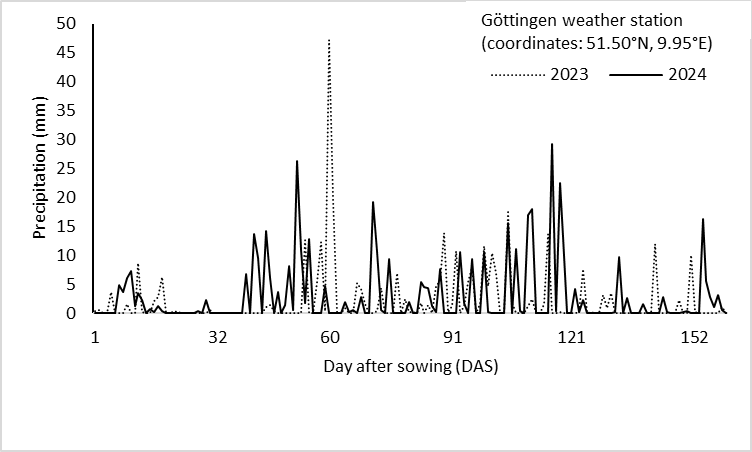


**Supplemental Figure 2**. Precipitation in Göttingen (mm) measured during the growing seasons 2023 and 2024.

**Average temperature, maximum temperature, minimum temperature, and precipitation during the growing season of 2023 as provided by the Deutscher Wetterdienst station in Göttingen.**

| **Day after sowing (DAS)** | **Precipitation (mm)** | **Average Temp.**  **(^0^C)** | **Maximum Temp.**  **(^0^C)** | **Minimum Temp.**  **(^0^C)** |
| --- | --- | --- | --- | --- |
| 1 | 0.3 | 9.8 | 13.1 | 4.5 |
| 2 | 0.5 | 6.0 | 9.8 | 0.6 |
| 3 | 0 | 3.7 | 8.9 | -1.9 |
| 4 | 0 | 6.4 | 13.1 | -0.3 |
| 5 | 3.6 | 10.1 | 14.4 | 6.5 |
| 6 | 0 | 10.3 | 12.5 | 2.4 |
| 7 | 0 | 8.2 | 16.1 | 0.5 |
| 8 | 0 | 11.2 | 19.6 | 1.3 |
| 9 | 1.6 | 9.3 | 11.9 | 2.8 |
| 10 | 0 | 7.4 | 15.5 | -0.9 |
| 11 | 0 | 10.6 | 18.8 | -0.2 |
| 12 | 8.6 | 12.7 | 19.2 | 4.4 |
| 13 | 0.2 | 13.8 | 19.2 | 8.9 |
| 14 | 0 | 10.5 | 14.4 | 7.3 |
| 15 | 0 | 12.1 | 18.6 | 5.2 |
| 16 | 2 | 14.7 | 20.7 | 7.4 |
| 17 | 2.8 | 13.8 | 19.1 | 11 |
| 18 | 6.3 | 12.8 | 15.2 | 9.7 |
| 19 | 0 | 14.3 | 20.5 | 9.1 |
| 20 | 0 | 13.5 | 20.5 | 5.7 |
| 21 | 0.3 | 11.8 | 20.4 | 5.4 |
| 22 | 0.1 | 12.4 | 19.8 | 5.4 |
| 23 | 0 | 9.9 | 14.4 | 2.9 |
| 24 | 0 | 8.6 | 13.6 | 1.5 |
| 25 | 0 | 8.5 | 15.6 | -0.6 |
| 26 | 0 | 11.5 | 18 | 2.6 |
| 27 | 0 | 13.4 | 17.2 | 8.5 |
| 28 | 0 | 17.5 | 24.7 | 7.8 |
| 29 | 0 | 19.2 | 25.9 | 11.9 |
| 30 | 0.5 | 12.3 | 15.3 | 5.3 |
| 31 | 0 | 9.1 | 14.7 | 1.3 |
| 32 | 0 | 10.8 | 17.9 | 3.6 |
| 33 | 0 | 11.5 | 18.1 | 3.7 |
| 34 | 0 | 11.9 | 20.2 | 2.6 |
| 35 | 0 | 14.5 | 23.6 | 2.6 |
| 36 | 0 | 14.6 | 20.5 | 6.4 |
| 37 | 0 | 13.6 | 19.7 | 5.9 |
| 38 | 0 | 15.6 | 24 | 5.4 |
| 39 | 0 | 12.4 | 18.2 | 5.1 |
| 40 | 0 | 12.7 | 18.2 | 8.3 |
| 41 | 0 | 13.5 | 20.3 | 3.9 |
| 42 | 0 | 13.7 | 22.3 | 2 |
| 43 | 0 | 14.6 | 24.2 | 3.1 |
| 44 | 1 | 15.0 | 21 | 5.4 |
| 45 | 1.4 | 18.3 | 23.1 | 13.8 |
| 46 | 0.3 | 18.7 | 25.2 | 12.2 |
| 47 | 0 | 18.9 | 26.8 | 9.2 |
| 48 | 0 | 18.8 | 27.3 | 9 |
| 49 | 0 | 18.7 | 26.5 | 9.4 |
| 50 | 0 | 17.4 | 25.3 | 7.5 |
| 51 | 0 | 17.7 | 25.5 | 6.6 |
| 52 | 0 | 16.1 | 22.3 | 8.2 |
| 53 | 0 | 17.0 | 24.1 | 6.2 |
| 54 | 12.6 | 15.4 | 24.7 | 8.3 |
| 55 | 0 | 18.1 | 26 | 9.4 |
| 56 | 0 | 19.9 | 29.1 | 8.9 |
| 57 | 5.3 | 22.8 | 28.9 | 16.4 |
| 58 | 12.3 | 21.8 | 28.2 | 16.9 |
| 59 | 0.3 | 21.1 | 27.6 | 15.8 |
| 60 | 47.2 | 20.2 | 28.3 | 15 |
| 61 | 19.5 | 16.9 | 21 | 11.8 |
| 62 | 0 | 18.8 | 26.9 | 10 |
| 63 | 0 | 20.9 | 28.8 | 12 |
| 64 | 0.9 | 20.3 | 27.4 | 11.5 |
| 65 | 0 | 15.7 | 20.9 | 9.4 |
| 66 | 0 | 16.5 | 23.5 | 7.7 |
| 67 | 5.2 | 19.4 | 24.7 | 14.7 |
| 68 | 4.1 | 15.8 | 19.7 | 9.5 |
| 69 | 1.6 | 16.1 | 20.9 | 8.8 |
| 70 | 0 | 16.8 | 20.4 | 11.8 |
| 71 | 0 | 16.3 | 20.5 | 12.1 |
| 72 | 0.4 | 17.3 | 22.9 | 11.8 |
| 73 | 4.4 | 17.2 | 20.6 | 10.9 |
| 74 | 0 | 16.4 | 23.4 | 9.1 |
| 75 | 0 | 18.1 | 27.8 | 7 |
| 76 | 0 | 21.2 | 31.4 | 8.8 |
| 77 | 7 | 23.5 | 33.8 | 11.7 |
| 78 | 0 | 19.9 | 25.7 | 13.1 |
| 79 | 2.4 | 22.9 | 32 | 11.2 |
| 80 | 0.1 | 20.9 | 25.4 | 12.4 |
| 81 | 0.2 | 17.7 | 24 | 11.8 |
| 82 | 0 | 19.1 | 26.8 | 9 |
| 83 | 1.7 | 21.8 | 31 | 13.3 |
| 84 | 0 | 20.4 | 26.2 | 12.1 |
| 85 | 1.4 | 17.8 | 25.5 | 9.4 |
| 86 | 0 | 16.9 | 24.7 | 7.7 |
| 87 | 4.6 | 16.5 | 23.9 | 9.8 |
| 88 | 5.6 | 16.7 | 22.7 | 10.6 |
| 89 | 13.9 | 14.5 | 21.3 | 10.6 |
| 90 | 0.1 | 16.2 | 23.9 | 9.1 |
| 91 | 1.3 | 19.0 | 23.4 | 13.4 |
| 92 | 10.7 | 19.4 | 27 | 14.4 |
| 93 | 0 | 15.6 | 20 | 9.5 |
| 94 | 1.2 | 14.0 | 21.9 | 7.2 |
| 95 | 5.4 | 15.0 | 18.7 | 9.9 |
| 96 | 8 | 19.8 | 25.1 | 16.2 |
| 97 | 0 | 19.5 | 25.9 | 14.3 |
| 98 | 1.5 | 17.5 | 22.1 | 12.9 |
| 99 | 11.5 | 17.6 | 20.2 | 14.6 |
| 100 | 4.6 | 17.6 | 22.6 | 15 |
| 101 | 10.3 | 17.0 | 22 | 13.1 |
| 102 | 7 | 16.9 | 20.4 | 15.3 |
| 103 | 0 | 16.9 | 22.2 | 10.6 |
| 104 | 0 | 16.8 | 24 | 9.5 |
| 105 | 17.4 | 16.0 | 22.9 | 13.2 |
| 106 | 1.8 | 14.5 | 18.3 | 9 |
| 107 | 0 | 14.3 | 19.5 | 10 |
| 108 | 0 | 15.2 | 21.3 | 10.1 |
| 109 | 0 | 16.1 | 23.4 | 7.6 |
| 110 | 1.2 | 19.5 | 29.3 | 8.8 |
| 111 | 2.5 | 20.3 | 26.9 | 14.7 |
| 112 | 0 | 18.6 | 24.9 | 12.8 |
| 113 | 0 | 21.0 | 28.9 | 12.2 |
| 114 | 1.5 | 21.3 | 29.6 | 15.3 |
| 115 | 13.7 | 20.8 | 25.7 | 17.4 |
| 116 | 0 | 19.7 | 25.2 | 17.1 |
| 117 | 0 | 23.3 | 30.9 | 17.2 |
| 118 | 0.1 | 23.9 | 34.6 | 16.5 |
| 119 | 0 | 22.9 | 29.9 | 17.1 |
| 120 | 0 | 22.4 | 28.6 | 16 |
| 121 | 0 | 20.0 | 26 | 12.9 |
| 122 | 0 | 18.6 | 26.3 | 10.7 |
| 123 | 0.1 | 20.2 | 29.2 | 10.8 |
| 124 | 7.6 | 19.4 | 23.4 | 15.5 |
| 125 | 0 | 18.1 | 22.7 | 11.8 |
| 126 | 0.2 | 16.2 | 21.9 | 9.1 |
| 127 | 0 | 14.3 | 19.9 | 7.7 |
| 128 | 0 | 15.1 | 20.6 | 8.6 |
| 129 | 3.2 | 14.1 | 21.3 | 6.9 |
| 130 | 0.9 | 13.7 | 18.3 | 9.3 |
| 131 | 3.5 | 14.2 | 18.6 | 8 |
| 132 | 0 | 16.7 | 23.1 | 12 |
| 133 | 0 | 15.8 | 21.5 | 9.3 |
| 134 | 0 | 16.1 | 25.3 | 6.5 |
| 135 | 0 | 20.1 | 29 | 10.9 |
| 136 | 0 | 20.2 | 30.2 | 10.2 |
| 137 | 0 | 20.1 | 30 | 9.3 |
| 138 | 0 | 19.7 | 30.8 | 10.1 |
| 139 | 0 | 19.2 | 29.9 | 9.3 |
| 140 | 0 | 19.9 | 30 | 10.9 |
| 141 | 0 | 20.7 | 30.5 | 11.7 |
| 142 | 11.9 | 20.3 | 27.3 | 13.7 |
| 143 | 0 | 16.6 | 22.6 | 8.7 |
| 144 | 0 | 12.2 | 18.9 | 6.1 |
| 145 | 0 | 14.3 | 23.1 | 6.4 |
| 146 | 0 | 17.4 | 26.5 | 8.6 |
| 147 | 0 | 20.6 | 28.7 | 14.1 |
| 148 | 2.3 | 19.9 | 23.3 | 14.4 |
| 149 | 0 | 16.6 | 19.7 | 14 |
| 150 | 0 | 18.6 | 23.9 | 13.3 |
| 151 | 10.1 | 17.7 | 22.1 | 11.3 |
| 152 | 0.2 | 15.4 | 19.9 | 11.9 |
| 153 | 0 | 13.5 | 17.6 | 10.1 |
| 154 | 0 | 14.0 | 19.9 | 9.5 |
| 155 | 0 | 13.6 | 21 | 7.4 |
| 156 | 0 | 13.7 | 24.3 | 4.3 |
| 157 | 0 | 16.3 | 25.2 | 8.3 |
| 158 | 0 | 17.9 | 26.3 | 11 |
| 159 | 1.1 | 18.2 | 25.6 | 12.7 |
| 160 | 0 | 13.1 | 19.1 | 9.2 |

**Average temperature, maximum temperature, minimum temperature, and precipitation during the growing season of 2024 as provided by the Deutscher Wetterdienst station in Göttingen.**

| **Day after sowing (DAS)** | **Precipitation (mm)** | **Average Temp.**  **(^0^C)** | **Maximum Temp.**  **(^0^C)** | **Minimum Temp.**  **(^0^C)** | |
| --- | --- | --- | --- | --- | --- |
| 1 | 0 | 17.3 | 25.1 | 11.4 |  |
| 2 | 0 | 12.8 | 18.8 | 8.8 |  |
| 3 | 0 | 9.2 | 14 | 2.4 |  |
| 4 | 0 | 9.8 | 17.4 | 1.1 |  |
| 5 | 0 | 14 | 19.2 | 9.9 |  |
| 6 | 0 | 16.1 | 21.3 | 9.9 |  |
| 7 | 4.8 | 13.3 | 17 | 8.3 |  |
| 8 | 3.7 | 9.1 | 14.9 | 2.4 |  |
| 9 | 6.1 | 6.2 | 9.1 | 4.1 |  |
| 10 | 7.3 | 5.8 | 10.7 | 3.7 |  |
| 11 | 1.3 | 6.2 | 12.3 | 2.8 |  |
| 12 | 3.5 | 5.8 | 8.8 | 4.4 |  |
| 13 | 2.2 | 5.8 | 9.6 | 2.8 |  |
| 14 | 0 | 4.2 | 8.5 | -1.2 |  |
| 15 | 0.7 | 2.4 | 8.3 | -1.9 |  |
| 16 | 0.1 | 3.6 | 10.5 | -3.4 |  |
| 17 | 1.2 | 5.1 | 9.4 | 3 |  |
| 18 | 0.4 | 5 | 9.9 | 0.8 |  |
| 19 | 0 | 8.6 | 14.2 | 1.9 |  |
| 20 | 0 | 10.8 | 19.9 | 0.4 |  |
| 21 | 0 | 13.7 | 20.4 | 5.1 |  |
| 22 | 0 | 13.3 | 20.8 | 6.4 |  |
| 23 | 0 | 16 | 25.2 | 7.1 |  |
| 24 | 0 | 18.1 | 25.2 | 7.8 |  |
| 25 | 0 | 17.6 | 23.8 | 8.5 |  |
| 26 | 0 | 12.9 | 15.2 | 9.7 |  |
| 27 | 0.4 | 12.5 | 16.8 | 9.3 |  |
| 28 | 0 | 13.4 | 17.7 | 8.4 |  |
| 29 | 2.3 | 12.9 | 17.7 | 8.2 |  |
| 30 | 0 | 11.9 | 15.2 | 6 |  |
| 31 | 0 | 12.1 | 19.1 | 4.4 |  |
| 32 | 0 | 12.1 | 21 | 2.4 |  |
| 33 | 0 | 13.9 | 22.9 | 4.7 |  |
| 34 | 0 | 15.1 | 23.1 | 6.3 |  |
| 35 | 0 | 15.1 | 21.8 | 5.6 |  |
| 36 | 0 | 18 | 24.8 | 9.6 |  |
| 37 | 0 | 18 | 24.2 | 8.5 |  |
| 38 | 0 | 18.7 | 24.5 | 9.3 |  |
| 39 | 6.8 | 16.1 | 20.2 | 9.7 |  |
| 40 | 0 | 15 | 20.9 | 8.5 |  |
| 41 | 13.7 | 14.1 | 19.1 | 11 |  |
| 42 | 9.5 | 14.2 | 19.2 | 10.3 |  |
| 43 | 0 | 15.6 | 21.9 | 10.3 |  |
| 44 | 14.1 | 16.5 | 23.8 | 8.4 |  |
| 45 | 6 | 15 | 18.6 | 10 |  |
| 46 | 0 | 15.3 | 22.1 | 9.3 |  |
| 47 | 3.7 | 13.9 | 20.1 | 8 |  |
| 48 | 0 | 16.7 | 22.7 | 11.4 |  |
| 49 | 1.4 | 17.3 | 24.1 | 9.3 |  |
| 50 | 8.2 | 16.8 | 23.4 | 11.2 |  |
| 51 | 0.6 | 14.4 | 18.4 | 11.9 |  |
| 52 | 26.2 | 15.3 | 18.7 | 11.6 |  |
| 53 | 10.9 | 13.5 | 16.4 | 10.7 |  |
| 54 | 1.7 | 15 | 19.4 | 11 |  |
| 55 | 12.8 | 16.7 | 23.3 | 13.1 |  |
| 56 | 0 | 14.8 | 16.5 | 10.2 |  |
| 57 | 0 | 12.4 | 15.3 | 10.5 |  |
| 58 | 0 | 14.8 | 21.1 | 10.7 |  |
| 59 | 4.6 | 13.9 | 17.7 | 8.4 |  |
| 60 | 0 | 13.9 | 22 | 6.1 |  |
| 61 | 0 | 13.9 | 20.3 | 7.8 |  |
| 62 | 0 | 16 | 23.6 | 6.5 |  |
| 63 | 0 | 14.7 | 20.2 | 7.4 |  |
| 64 | 1.9 | 11.8 | 16.6 | 5.5 |  |
| 65 | 0.1 | 11.5 | 16.8 | 6.3 |  |
| 66 | 0.6 | 10.5 | 16.7 | 4.9 |  |
| 67 | 0 | 12.1 | 18.9 | 4.9 |  |
| 68 | 2.8 | 14.1 | 19.2 | 8.5 |  |
| 69 | 0 | 15.5 | 20.1 | 8.5 |  |
| 70 | 0 | 16.2 | 21.1 | 10.5 |  |
| 71 | 19.2 | 16.6 | 22 | 13 |  |
| 72 | 10.6 | 18.5 | 24.7 | 13.1 |  |
| 73 | 0.5 | 14.9 | 20 | 9.5 |  |
| 74 | 0 | 15.8 | 20.9 | 8.8 |  |
| 75 | 9.3 | 19.3 | 24.2 | 15.1 |  |
| 76 | 0 | 17.1 | 23 | 11.7 |  |
| 77 | 0 | 17 | 23.5 | 9.7 |  |
| 78 | 0 | 18.4 | 26 | 9.8 |  |
| 79 | 0 | 19.7 | 26.8 | 10.6 |  |
| 80 | 1.9 | 22.4 | 29.7 | 11.9 |  |
| 81 | NA | 22.1 | 29.6 | 16.3 |  |
| 82 | 0 | 20.4 | 25.7 | 11.3 |  |
| 83 | 5.3 | 20.7 | 29 | 9.9 |  |
| 84 | 4.5 | 18.3 | 23.2 | 15.6 |  |
| 85 | 4.3 | 15.2 | 19.7 | 12.8 |  |
| 86 | 1.2 | 13.9 | 17.2 | 10.5 |  |
| 87 | 0.1 | 13.1 | 16.1 | 10.2 |  |
| 88 | 7.6 | 14.1 | 19.8 | 8.7 |  |
| 89 | 0 | 15.1 | 19.1 | 9.9 |  |
| 90 | 0 | 18.9 | 27.3 | 11.7 |  |
| 91 | 0 | 15.7 | 22.1 | 7.8 |  |
| 92 | 0 | 17.5 | 25.8 | 8.2 |  |
| 93 | 10.6 | 22.7 | 31.3 | 12.6 |  |
| 94 | 1.7 | 20.7 | 26 | 16.9 |  |
| 95 | 0 | 20.2 | 25.5 | 15.5 |  |
| 96 | 9.3 | 19 | 27.3 | 13.6 |  |
| 97 | 0 | 16.4 | 22.3 | 10.1 |  |
| 98 | 0 | 17.2 | 24.2 | 8.5 |  |
| 99 | 10.6 | 20.1 | 27.8 | 10.7 |  |
| 100 | 0.1 | 19.8 | 25.4 | 13.6 |  |
| 101 | 0 | 17.7 | 22.8 | 11.8 |  |
| 102 | 0 | 19.1 | 27.2 | 10.2 |  |
| 103 | 0 | 20.3 | 28.3 | 11.1 |  |
| 104 | 0 | 22.1 | 31.2 | 12.8 |  |
| 105 | 15.5 | 21.2 | 31.5 | 15 |  |
| 106 | 0 | 20.2 | 24.6 | 16.5 |  |
| 107 | 11.1 | 19.1 | 27.5 | 14 |  |
| 108 | 0.3 | 17 | 22.2 | 10.7 |  |
| 109 | 0 | 17.3 | 24.6 | 9.1 |  |
| 110 | 16.9 | 19 | 24.5 | 15 |  |
| 111 | 17.9 | 19.6 | 23.7 | 17.5 |  |
| 112 | 0 | 18.9 | 24.3 | 12.2 |  |
| 113 | 0 | 17.9 | 26 | 10.4 |  |
| 114 | 0 | 20.6 | 30.1 | 10.3 |  |
| 115 | 0 | 21.1 | 29.7 | 13.9 |  |
| 116 | 29.2 | 20.6 | 29 | 13.8 |  |
| 117 | 0.3 | 19.5 | 24.3 | 14.7 |  |
| 118 | 22.5 | 19.6 | 27.4 | 12.7 |  |
| 119 | 13.1 | 18.3 | 21.7 | 13.9 |  |
| 120 | 0 | 18.1 | 23 | 13.3 |  |
| 121 | 0 | 20.5 | 28.2 | 12 |  |
| 122 | 4.2 | 20.5 | 28 | 13.6 |  |
| 123 | 0.1 | 17.7 | 23.3 | 12.7 |  |
| 124 | 2.3 | 18.6 | 25.5 | 12.2 |  |
| 125 | 0 | 19.8 | 26.5 | 12.8 |  |
| 126 | 0 | 20.6 | 27 | 14.2 |  |
| 127 | 0 | 22.2 | 30.7 | 11.9 |  |
| 128 | 0 | 25.5 | 33.5 | 18.2 |  |
| 129 | 0 | 23.6 | 29.2 | 19 |  |
| 130 | 0 | 22 | 27.6 | 16.2 |  |
| 131 | 0 | 21.5 | 28.5 | 15.4 |  |
| 132 | 0.1 | 21 | 26 | 17.7 |  |
| 133 | 9.7 | 17.8 | 22.1 | 15.1 |  |
| 134 | 0 | 16.9 | 22.3 | 11.9 |  |
| 135 | 2.6 | 19 | 26.7 | 9.8 |  |
| 136 | 0 | 17.1 | 20.9 | 10.7 |  |
| 137 | 0 | 17.5 | 23.3 | 10.7 |  |
| 138 | 0 | 21.4 | 27.9 | 15.2 |  |
| 139 | 1.6 | 24.2 | 31.5 | 17.4 |  |
| 140 | 0 | 17.7 | 22.5 | 9.9 |  |
| 141 | 0 | 15.4 | 23.6 | 7.3 |  |
| 142 | 0 | 17.2 | 26.4 | 7.1 |  |
| 143 | 0 | 20.3 | 30.8 | 9.6 |  |
| 144 | 2.8 | 22.7 | 32.8 | 14.4 |  |
| 145 | 0.1 | 18.6 | 23.8 | 16.5 |  |
| 146 | 0 | 19 | 24.3 | 15 |  |
| 147 | 0 | 20.5 | 27.9 | 12.5 |  |
| 148 | 0 | 21.6 | 29.6 | 13.3 |  |
| 149 | 0.2 | 22.4 | 29.5 | 14.8 |  |
| 150 | 0.4 | 21.7 | 27.3 | 16.6 |  |
| 151 | 0 | 24.9 | 32.5 | 18.5 |  |
| 152 | 0 | 23.5 | 28.6 | 19.1 |  |
| 153 | 0 | 21.6 | 30.5 | 14.1 |  |
| 154 | 16.3 | 19.2 | 27.6 | 12.3 |  |
| 155 | 5.6 | 16.7 | 20.8 | 15.2 |  |
| 156 | 2.8 | 15.3 | 18.5 | 10.7 |  |
| 157 | 1 | 12.3 | 16.6 | 5.9 |  |
| 158 | 3.2 | 8.8 | 14 | 4.2 |  |
| 159 | 0.5 | 9.8 | 14.5 | 4.1 |  |
| 160 | 0 | 10.5 | 16.1 | 4.8 |  |
